# Torsional nystagmus and otolith dumping effects investigated by head circumduction

**DOI:** 10.1152/jn.00570.2024

**Published:** 2025-03-28

**Authors:** Michele Corrado, Bianca Nicklen, Yuxiao Li, Toby Jack Ellmers, Adolfo Miguel Bronstein

**Affiliations:** 1 Department of Brain and Behavioural Sciences, University of Pavia, Pavia, Italy; 2 Movement Analysis Research Section, IRCCS Mondino Foundation, Pavia, Italy; 3 Department of Brain Sciences, Imperial College, London, United Kingdom

**Keywords:** eye movements, post-rotational, semicircular canals, vestibulo-ocular reflex, vestibular

## Abstract

This study introduces a novel paradigm to induce the torsional vestibulo-ocular reflex (VOR) using head circumduction - a circular motion of the head combining extension, lateral rotation and flexion of the neck. The aims were to (i) evaluate the reliability of this manoeuvre in generating torsional nystagmus and (ii) explore the impact of post-rotational head tilt on induced VOR responses. Fourteen healthy participants (age = 27.3±5.3 years) were tested using a 3D eye-tracker to record eye movements induced by head circumduction, performed at a frequency of 0.75 Hz. Participants were recorded on stopping in either a head-up or head-down condition. All participants showed robust post-rotational torsional nystagmus. However, this was significantly shorter during head-up (average duration = 10.7±2.4 s, with a time constant of 4.1±1.1 s) compared to head-down (average duration = 15.7±3.7 s, with a time constant of 7.2±2.5 s; p = 0.0001). Vertical nystagmus was also observed in most participants, which was either disconjugate or overtly skewed. The shortening of torsional nystagmus duration and time constant in the head-up position supports (i) a role for the velocity storage mechanism in the torsional VOR (which was previously disputed) and (ii) the existence of otolith dumping effects in the torsional VOR. In addition, the vertical ocular findings during the stopping response confirm that skewed eye movements can be generated by vertical semicircular canal activity. Our findings support the feasibility of head circumduction as a simple method for assessing semicircular and otolith effects on the torsional VOR.

## Introduction

The vestibulo-ocular reflex (VOR) generates dynamic, slow-phase compensatory eye movements aimed at stabilizing gaze during head motion (1, 2). These eye movements occur in the horizontal, vertical, and torsional planes, depending mainly on the stimulated semicircular canals (SCCs). Notably, VOR gain varies across these three planes, with higher gain in the horizontal and vertical planes, and lower gain in the torsional plane (3). In humans, VOR-generated eye movements can be evaluated during short, fast acceleration head rotations (4), or prolonged constant velocity stimuli. In the latter case, a post-rotational opposite, and normally symmetric, nystagmic response can be observed (5).

Both during and immediately following constant velocity rotations, VOR responses will typically exhibit an exponential decay, reflecting the SCCs’ progressive decline in activity (5). However, the time constants (TCs) of these decaying responses differ across planes and are influenced by various factors, such as head positioning (6). Importantly, they do not only reflect the TCs of the vestibular nerve or the SCCs involved (5, 7). For instance, during yaw rotations, the TC of the horizontal nystagmus is approximately 14-18 seconds (6), which is longer than the vestibular nerve’s expected TC (4-8 s). This discrepancy is believed to result from the input of a central velocity storage mechanism (8). Conversely, vertical and torsional nystagmus exhibit shorter TCs (5), closer to the expected values for the vestibular nerve, which led to the hypothesis that the velocity storage has little or no role in these planes (5, 9).

Additionally, post-rotational forward head tilt can strongly shorten the TC of horizontal nystagmus (6), which is thought to be evidence of velocity storage modulation mediated by otolithic tone, referred as ‘otolith dump’ (8, 10, 11). To our knowledge, no human studies have assessed the role of post-rotational head tilt influence on the torsional VOR decay, although one study has investigated a putative velocity storage modulation in the vertical VOR (12). Interestingly, studies performed on monkeys found an effect of post-rotational head tilt on the TC of torsional nystagmus (7, 13). These differences across the three planes of eye movements underscore the role of studying such VOR-generated responses, both to assess the physiological bases of peripheral and central vestibular processing as well as understanding the relatively neglected clinical conditions incorporating torsional nystagmus. However, evaluating the torsional VOR with long rotational stimuli necessitates the use of complex multiaxial rotatory frames (3, 5, 7, 13), or rotating couches (9, 14, 15). As such, literature assessing this kind of induced torsional VOR is limited to few studies.

Given these limitations, we aimed to develop a novel paradigm for inducing torsional VOR in humans that is both simple and practical. Specifically, head circumduction, which involves a combination of extension, lateral rotation, and flexion in a circular motion, is a well-established method for neck rehabilitation and assessment (16–18). However, continuous head circumduction is rarely performed because clinical observations reveal that this complex – albeit natural head motion – often induces vertigo and unsteadiness. Based on these observations, we hypothesized that head circumduction could be a potent inducer of torsional nystagmus. Moreover, if this were the case, examining the torsional VOR response during post rotational (“stopping”) responses with the head up or down would place the expected semicircular canal response either neutral (head down) or in conflict (head up) with the gravity vector. Any significant shortening of the post-rotational nystagmic response with the head up would imply, according to accepted knowledge (7, 8, 13), an otolith dumping effect, hence the existence of an operational velocity storage mechanism in the vertical semicircular canal system.

Consequently, we planned to investigate the eye movements of the stopping response induced by this paradigm. Our aims were to therefore determine: (1) whether circumduction head movements reliably induce torsional nystagmus, validating it as a stimulation technique for the torsional VOR system, and (2) to explore the effect of head reorientation on the decay pattern of the stopping response, therefore assessing a putative role of otolith dumping on the human velocity storage mechanism in the torsional plane.

## Materials and Methods

### Study population

For the experiment, we enrolled healthy participants. The inclusion criteria were aged between 20-40 years and the ability to comply with the study protocol. Exclusion criteria included the presence of cervical or neuro-oto-ophthalmological conditions, and the use of alcohol and/or other CNS acting drugs in the 48 hours before the study. All participants agreeing to participate in the study signed an informed consent before any procedure. The study protocol received approval by the Essex Research Ethics Committee (REC Reference: 23/EE/0176) and was conducted in accordance with the recommendations adopted by the World Medical Assembly (Helsinki 1964 and later revisions). Before performing the experimental paradigm, all participants received a comprehensive oculo-vestibular examination, which included an assessment of gaze, cover test, spontaneous and positional nystagmus, saccadic and pursuit eye movements as well as a horizontal and vertical head impulse test.

### Experimental paradigm

The main study procedures were carried out in a single session. First, after screening for inclusion/exclusion criteria and signing of informed consent, participants were instructed on how to perform the experimental paradigm.

**Figure 1:**
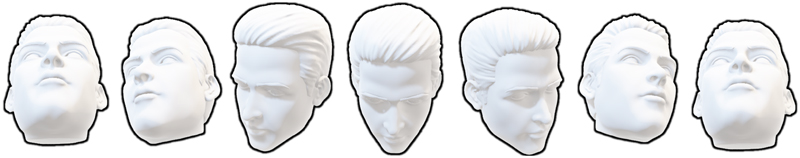
Schematic representation of a right circumduction. Schematic representation of a participant’s right head circumduction. From left to right: participants started the circumduction task while looking up, then rotated their head right while going downward. Subsequently, participants went towards their left shoulder. While turned towards their left shoulder, participants went upward and would end one circumduction going back to the starting point. For each task, participants would perform 20 head circumduction at a metronome controlled speed of 45 beats-per-minute (one complete circumduction every beat).

The experimental paradigm required the participants to perform a head circumduction task. Namely, the participants performed unidirectional, continuous circular head rotation movements (Figure 1) at a metronome-controlled frequency of 45 beats-per-minute, with each beat consisting in a complete circular movement with their head, thus resulting in a frequency of 0.75 Hz. Rotations were performed in complete darkness, without a fixation point.

Each task consisted of 20 complete rotations, for a total duration of 26.6 seconds. At the end of each task, participants were instructed to stay still with their eyes open and head upright (HU) in a neutral position, or with their head (face) facing down (HD). We defined right circumduction (RC) tasks as those during which the participant’s head rotated from top-to-bottom while turned towards the right shoulder, and left circumduction (LC) for the opposite movement (Figure 1). From the participant’s point of view RC resulted in a clockwise rotatory head movement; from the experimenter’s point of view this resulted in a counterclockwise head movement. Please see the supplementary materials for an exemplificative video showing the circumduction task and the stopping response. The participants, always seated on a sturdy chair, were instructed to move their head both laterally and up/down around 30-45°, we instructed the subjects to prioritize a continuous, smooth movement rather than focusing on excessive degrees of flexion/rotation. For the post-rotational head down condition, they were also instructed to reach the head down position by flexing the head roughly 90° forward, pointing the nose towards the floor, without resting on a surface.

Once the participants were considered capable of performing the experiment, they were seated in a windowless dark room, while wearing the 3D eye-tracker device (see below section for details). Participants were able to hear the metronome beats from a speaker and were visually monitored by an investigator via an infrared camera to assess for optimal compliance. During each task, immediately on stopping, eye movements were recorded (see below) and monitored in real-time by the investigator. Each recording lasted for a minimum of 30 seconds after the end of any screen-visible post-rotational nystagmus.

Each participant performed both right and left circumduction, while stopping both in the head up and down conditions; each task was repeated twice, for a total of 8 tasks. All tasks were performed in a randomized order via a latin square randomization design. At the end of each task, participants were systematically asked to describe the presence of vestibular symptoms (19), namely spinning or non-spinning (e.g. rocking) vertigo and dizziness. We also assessed for nausea or pain occurring either during the head circumduction or on stopping. Between each task, participants were allowed to rest at least 5 minutes after the end of any vestibular symptoms.

We planned to stop the protocol if any adverse event or discomfort with the procedure were to occur (such as disabling nausea or neck pain), although this was never necessary. The tasks were repeated if slippage of the eye tracker goggles occurred during rotations.

As a secondary experiment, we checked for response consistency and the effect of a more prolonged stimulus in a subgroup of participants, 4 weeks after the first evaluation. The procedure was repeated as above with the only difference of prolonging the head circumduction to 45 rotations, for a total of 59.9 s for each task.

### Eye movements evaluation

For the off-line evaluation of eye tracking, data were collected from both eyes during all the tasks. The video-oculography was performed with a 3D eye-tracker device (Chronos Vision C-ETD, 2014, Germany). The head mounted unit has two digital infrared cameras and communicates with a dedicated system unit for image processing. Image recording was performed at a frequency of 100Hz. Resolution for eye movements in the vertical, horizontal and torsional plane is <0.05°, with a noise of <0.02° root-mean-square. The system software (Chronos Vision C-ETD, 2014) was used to perform pupil tracking for horizontal/vertical eye movements, and the iris segment tracking algorithm for the torsional component. Eye movement calibration with targets placed 10 degrees apart in the horizontal and vertical plane was performed before each task; torsional calibration was software derived. Eye tracking data were low pass filtered at 10 Hz and analysed using a custom-written script in MATLAB™ (R2024a, The MathWorks, Inc.), to see the slope of slow-phase decay over time, the signal has been smoothed and saccades removed by using the Savitzky-Golay polynomial filter with the Data Cleaner MATLAB™ tool. For each post-rotational response, we analysed the type and direction of nystagmus in the three planes (torsional, vertical, and horizontal), its total duration (set from the onset of the first clearly configured nystagmic beat to the end of the response, when the slow phase trace was flat, and no quick components were visible), the peak slow-phase velocity (SPV) and its time constant (i.e. the time in which SPV reaches 37% of its initial, peak value).

We analysed the data from each eye individually, which were subsequently presented merged if no asymmetries were noted. We expected from previous literature, which was confirmed herein, to observe symmetrical features for torsional and horizontal nystagmus but not for vertical eye movements (9). The latter could present a disconjugate or “skew” response; thus, we present vertical data for each individual eye. Additionally, for the vertical component, we calculated the cumulative slow-phase vertical divergence (14), defined as the difference between the two eyes in absolute displacement (°) during the slow-phase of the nystagmus response.

### Head motion assessment

Exploratively, and in order to assess the head dynamics during the task more precisely, an inertial sensor (Opal V2R^®^, APDM) was placed above the nasion, mounted orthogonally on the goggle’s frame, for a subset of 5 participants. The sensor incorporates a triaxial accelerometer (16 bits/axis), triaxial magnetometer (13 bits), and triaxial gyroscope (16 bits/axis) recorded at 128 Hz. The sensor records linear acceleration and angular velocity along the three axes: anteroposterior (roll plane angular velocity), mediolateral (pitch plane angular velocity) and vertical (yaw plane angular velocity). For each task, gyroscopic data were low pass filtered at 3 Hz and analysed using a custom-written script in MATLAB™. Based on the roll plane head angular velocity, we were then able to report torsional VOR gain, by calculating the ratio between the peak SPV and the peak roll plane angular velocity for every task.

### Statistical analysis

Statistical analyses were carried out with JASP software (JASP Team 2024 - JASP Version 0.18.3). Descriptive statistics of results variables have been presented as mean ± standard deviation. After verifying the normality of the distributions with the Shapiro – Wilk test, which revealed a non-normal distribution of some variables (such as the cumulative slow-phase divergence and time constants), a Wilcoxon signed-rank test was carried out to assess differences in post-rotational nystagmus features (e.g. left vs. right circumduction tasks and head up vs down conditions). Additional subgroups differences were assessed via Mann-Whitney test.

For the results, nystagmus velocity data are presented in degrees/seconds (°/s), with positive values indicating either a rightward or upward ocular displacement, and the opposite for negative values. For torsional coordinates rightwards means top of the eye or head moving from the resting position towards the right shoulder. Gyroscopic signals are expressed in the three planes of rotation: pitch (antero-posterior), roll (frontal), and yaw (medio-lateral). Velocity data are presented in °/s, with positive values indicating either a rightward or upward displacement, and the opposite for negative values.

## Results

### Demographic features

We screened 16 participants; one was excluded for lack of optimal compliance with the head movement required, as the participant would not perform a continuous movement and frequently paused following each rotation. Of the 15 enrolled participants, one was excluded during the data analysis for poor iris tracking due to droopy eyelids, a well-recognized limitation for video-oculography measurements (20). The final dataset consisted of 14 healthy participants (4 males, 28.6%, mean age 27.3±5.3 years).

All 14 participants successfully performed the eight experimental tasks, which were well tolerated with no reported symptoms during the actual head circumduction. Immediately on stopping, all participants reported mild vestibular and/or vegetative symptoms: non-spinning vertigo (11 participants, 78.6%), spinning vertigo (3 participants, 21.4%), nausea (4 participants, 28.6%).

No subjective differences were noted on stopping between left and right circumduction. While in the head down condition, 9 (64.3%) participants reported that the symptoms were less intense, in 2 participants (14.3%) they worsened, 3 (21.4%) participants reported no change in the HD condition (Table 1).

### Post-rotational torsional nystagmus features

**Figure 2:**
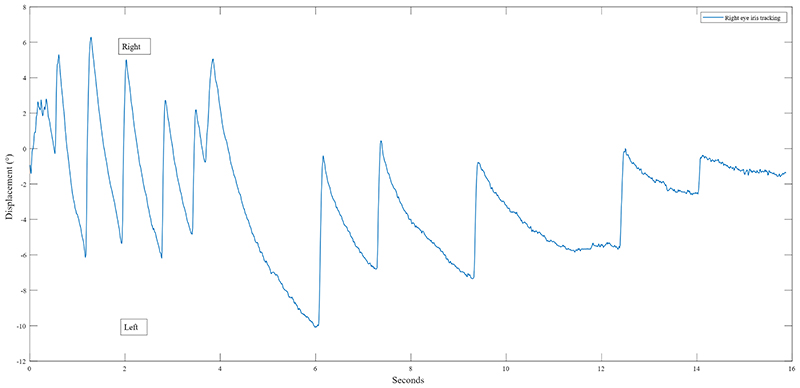
Representative participant’s right-beating post-rotational torsional nystagmus. Representative participant’s eye tracking plot of a post-rotational right-beating torsional nystagmus after a right circumduction task. The blue line represents the torsional displacement (°) of the right eye of the participant across time. Figure legend: ° = degrees.

All participants (100%) showed a robust post-rotational torsional nystagmus, which was consistently right-beating (upper poles beating towards the participants’ right shoulder) after the right circumduction tasks and left-beating (upper poles beating towards the participants’ left shoulder) after the left circumduction tasks (Figure 2). Since the comparison between right and left tasks did not show significant differences (p>0.091 for all variables), we present the averaged absolute data merged from both sides of circumduction. In the supplementary tables we provided data for individual sides of circumduction (supplementary table 1).

### Head Up and Down conditions

**Figure 3:**
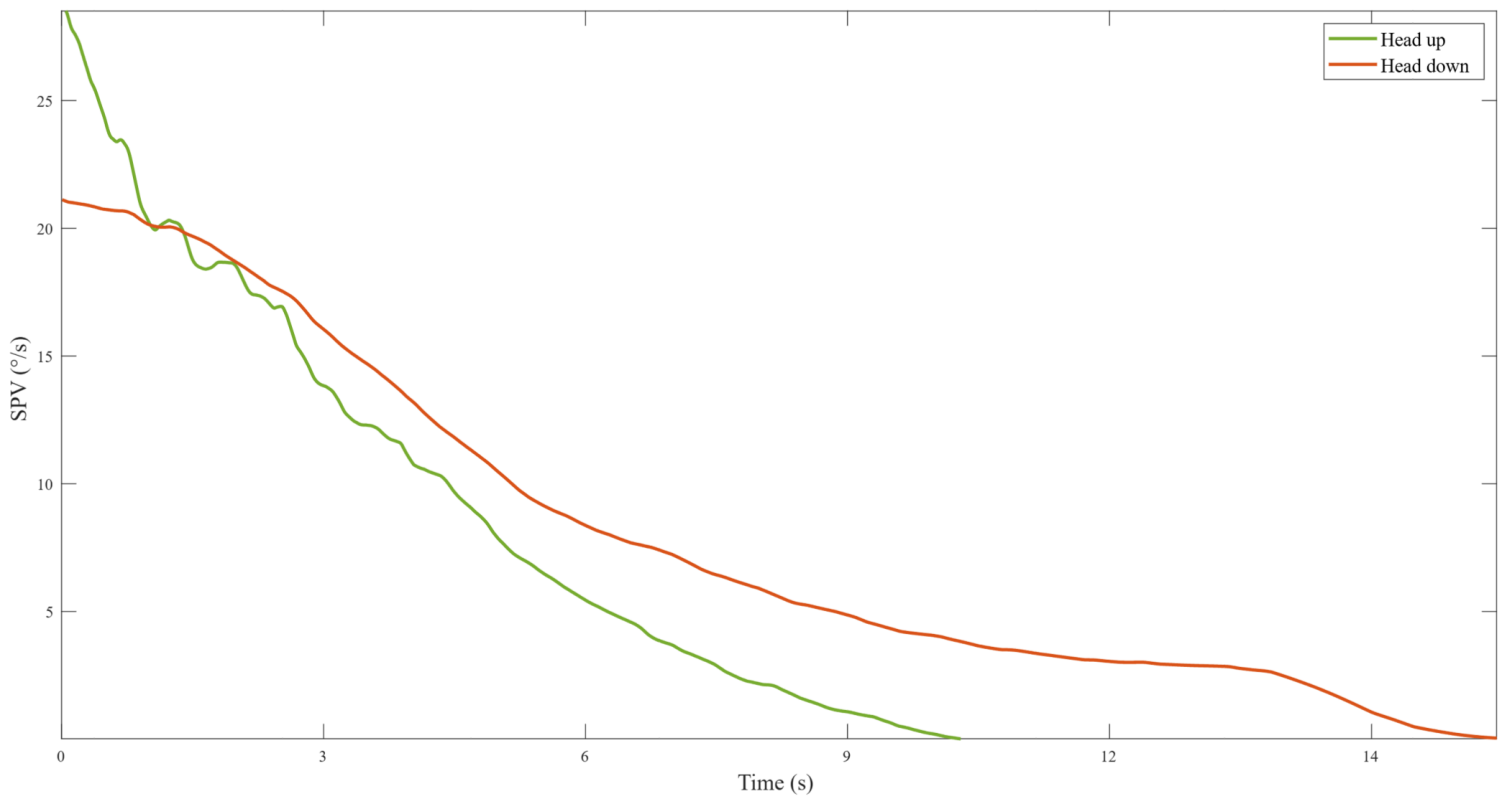
Representative participant’s time decay of the torsional SPV. Representative participant’s plot of the average post-rotational slow-phase velocity in the two conditions across time, depicting the exponential time-decay of the torsional response. Saccades have been filtered out and signal has been smoothed. The green line represents the slow-phase velocity of the torsional nystagmus in the head up condition, while the orange line represents it in the head down condition. Figure legend: SPV = slow-phase velocity, °/s = degrees/seconds.

**Figure 4:**
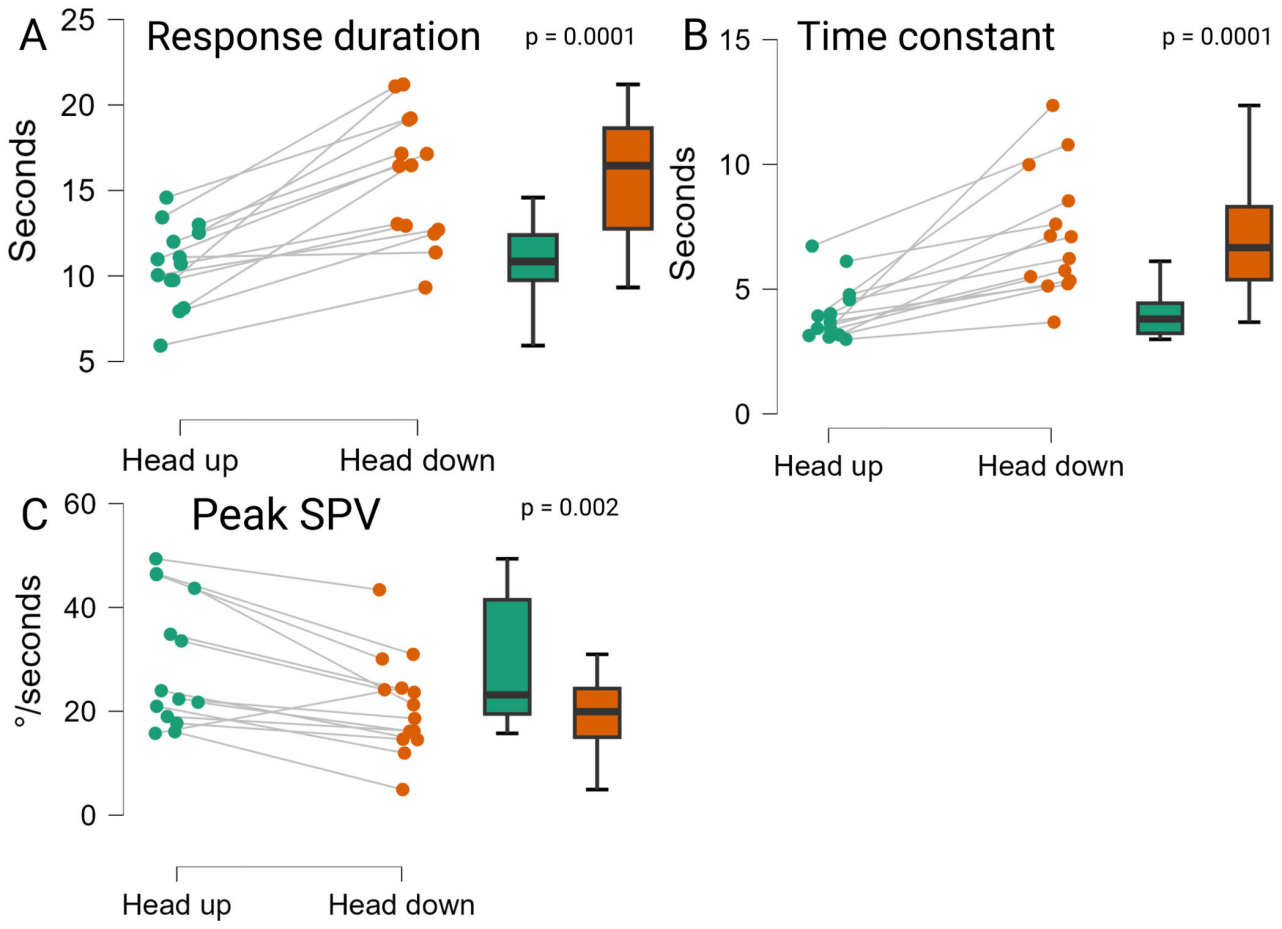
Comparison between head up and down conditions of torsional nystagmus features. Paired comparison (Wilcoxon signed-rank test) of torsional nystagmus features between the participants in head up (green) and head down (orange) post-rotational conditions. Right and left circumduction tasks have been merged. Each dot represents a single participant. **A:** response duration. **B:** time constant of response. **C:** peak slow-phase velocity.

The average duration of response in the HU was 10.7±2.4s, with an average time constant (TC) of 4.1±1.1s. During HD, the average duration of response was longer, lasting 15.7±3.7s with a TC of 7.2±2.5s (Figure 3). At the statistical comparison between head up and down conditions, the prolongation of response duration and time constant in HD was statistically significant (p=0.0001 for both, Figure 4 A, B). Peak SPV in the HU post-rotational conditions was 29.4±12.5°/s. In the HD post-rotational conditions, mean peak SPV was 21.1±9.5°/s. The peak SPV was significantly slower in HD (p=0.002) (Figure 4, C).

### Vertical nystagmus features: post-rotational disconjugacy and skew deviation

Since the vertical features were symmetrical between the two sides of rotation (p>0.77 for all comparisons), we present the averaged data merged from both sides of circumduction. In supplementary table 2 we provided data for individual sides of circumduction.

**Figure 5:**
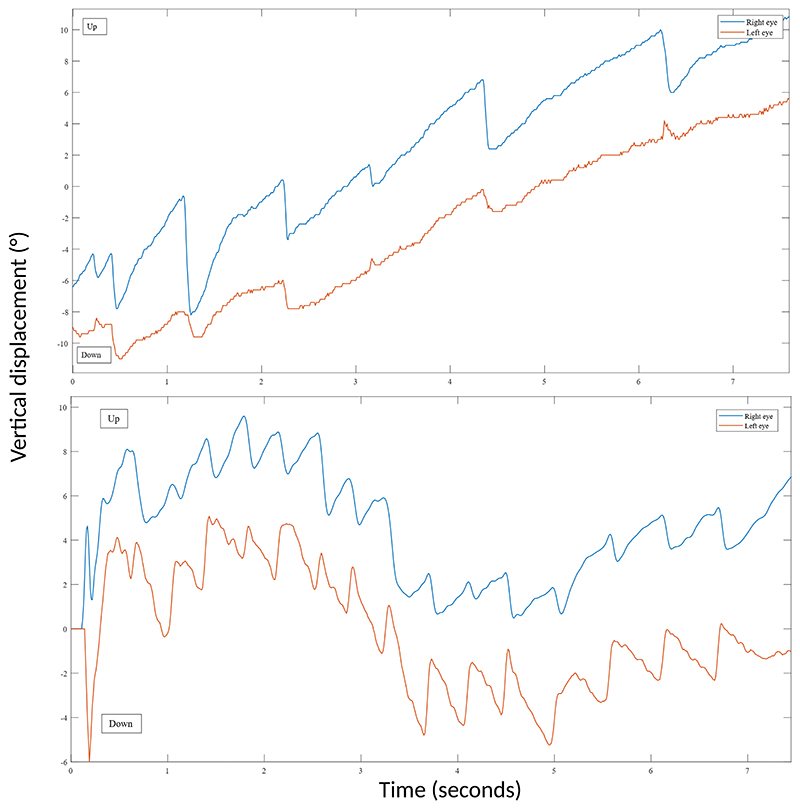
Representative participants’ post-rotational vertical nystagmus. The blue line represents the vertical displacement (°) of the right eye, while the orange line represents the vertical displacement of the left eye of the participants across time. **Top graph:** Representative eye tracking plot of a post-rotational bilateral down-beating nystagmus after a right circumduction task. Note the disconjugacy between the two eyes. **Bottom graph:** representative eye tracking plot of a post-rotational vertical skew response after a right circumduction task. Note the opposite polarity of the two eyes. Figure legend: ° = degrees, s = seconds

Ten participants (71.4%) showed, concomitantly with the torsional nystagmus, a post-rotational down-beating nystagmus (Figure 5), which was disconjugate between the two eyes: the down-beating fast phase of the extorting eye (upper pole of the eye tilting towards the participant’s temporal bone) was larger than the fast phase of the intorting eye (upper pole tilting towards the participant’s nose). This was because the vertical upward peak SPV of the extorting eye in the HU condition was 8.9±4.7°/s, while in the intorting eye it was 3.6±3.5°/s (p=0.0005). In the HD condition, the vertical upward SPV of the extorting eye was 9.1±4.3°/s, while in the intorting eye was 4.7±3.0°/s (p=0.0005). This vertical disconjugacy resulted in a cumulative slow phase displacement of 14.4±7.5° and of 16.8±9.7° in the HU and HD conditions, respectively (Table 2). There were no statistically significant differences between HU/HD conditions (p=0.301, Table 2).

Notably, three participants (21.4%) showed a post-rotational vertical skew response, meaning that the intorting eye during the fast phase was up-beating, while the extorting eye was down-beating (Figure 5). This vertical dissociation, along with the torsional component, resembles the clinical description of “hemi-seesaw nystagmus”, where the torsion is conjugate and the vertical components are in opposite directions (21). For these three participants, in the HU condition the cumulative SPD was 35.7±39.8° and in the HD condition was 45.5±34.6° (Table 2). There were no statistically significant differences between the two conditions (p=0.750). Finally, 1 participant (7.14%) did not show any vertical eye movements.

### Post-rotational horizontal nystagmus features

Since the horizontal features were not statistically different between the two sides (p>0.305 for all), we present the averaged absolute data merged from both sides of circumduction. In the supplementary tables we provided data for individual sides of circumduction (Supplementary table 3).

Overall, horizontal eye movement components were weak and inconsistent. In 7 participants (50%) a horizontal post-rotational nystagmus, beating toward the same side of the torsional component was noted. In the HU for these participants, the peak SPV was 8.5±4.0°/s in the HD was 4.7±1.5°/s. In 2 participants (14.3%) the post-rotational horizontal nystagmus was present and consistently beating in the opposite direction of the torsional component. In the HU for these participants, the peak SPV was 5.7±4.7°/s, while in the HD was 5.8±3.5°/s. In 3 participants (21.4%), a post-rotational horizontal nystagmus was present and always beating toward the left side, regardless of the task. In these participants, during HU, the peak SPV was 4.9±1.3°/s, while in HD was 6.6±3.8°/s. No significant differences emerged between HU and HD conditions (p>0.500 for all comparisons). Finally, 2 participants (14.3%) did not show any horizontal component, regardless of the task.

### Additional observations

As additional findings, 2 participants (14.3%) showed a persistent vertical nystagmus in the dark. Of these, one participant showed a persistent up-beating nystagmus only present in the HU condition, regardless of the task (SPV of 2.5±1.1°/s). The other participant showed a persistent down-beating nystagmus only present in the HD condition, regardless of the task (SPV of 2.6±0.4°/s). These were evident after a short latency from the end of the post-rotational response (0.8±0.2s). Similar findings are observed in normal subjects (22).

As we were focused on the stopping response, we did not record the eye movements during the actual head rotation, however, we noticed online on the VOG screen that the eye movements generally mirrored the head circumduction. For instance, during right circumduction, the eyes would also perform a circular movement, being eccentric in the orbit, going from top to bottom while looking toward the right shoulder of the subjects, as in the “look where you’re going” strategy (23). Such large gaze shifts during head movements have been noted in previous research, and imply a VOR suppression strategy during active motion (24, 25).

### Head motion analysis

Five participants performed the tasks while also wearing an inertial measurement unit (IMU). Based on the IMU data, we confirmed that all participants recorded performed 20 complete head circumduction movements for each task, for an average duration in HU of 27.5±0.9s. In HD, the tasks lasted 26.7±0.5s. To reach the HD position at the end of the tasks, participants took an average of 1.0±0.1s.

**Figure 6:**
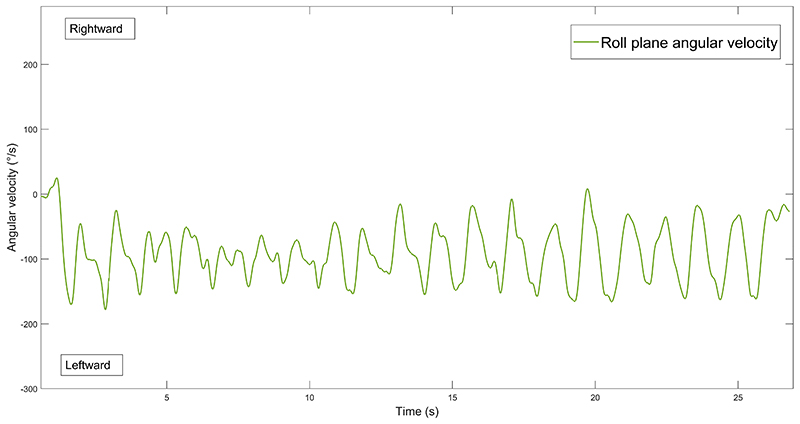
Exemplificative participant’s right circumduction roll angular velocity. Exemplificative plot of a participant’s roll plane angular velocity during the right circumduction. Note the continuous overall leftward roll velocity. Figure legend: °/s = degrees/seconds; s = seconds.

Data from the angular velocity recordings revealed a quasi-sinusoidal oscillation pattern in pitch and yaw plane, which was symmetrical, without statistically significant differences when comparing the two sides of circumduction (p>0.156 for all comparisons). Being oscillatory, velocity signals in pitch and yaw cancel each other thus reaching slow net average velocity (on average 1.2±4.6°/s and 1.7±5.1°/s for pitch and yaw, respectively). We reported subsequently the roll angular velocity data but in supplementary materials velocity data for pitch and yaw can be found (Supplementary figure 1 and 2). In the roll plane, we found a consistent, unidirectional velocity profile (Figure 6). Thus, we present the roll data for single sides of circumduction.

In the roll plane angular velocity, for the HU-RC condition, the peak rightward velocity was 31.7±21.0°/s, while the peak leftward velocity was -127.8±73.8°/s, the overall average velocity was -42.6±24.4°/s (meaning an overall leftward roll motion). In HU-LC, the peak rightward velocity was 116.0±75.2°/s, while the peak leftward velocity was -23.8±33.8°/s, the overall average velocity was 35.3±15.8°/s (meaning an overall rightward roll motion). For the HD condition, in RC, the peak rightward velocity was 27.2±22.0°/s, while the peak leftward velocity was -112.5±63.4°/s, the overall average velocity was -37.3±18.8°/s. In HD-LC, the peak rightward velocity was 150.7±84.7°/s, while the peak leftward velocity was -57.5±37.9°/s, the overall average velocity was 31.4±10.5°/s. We did not find any statistically significant differences, when comparing the gyroscopic data between the tasks performed in HU and HD conditions (p>0.136 for all comparisons). Regarding the torsional VOR gain, we calculated the gain on a participant-by-participant basis, by computing the ratio between the peak torsional SPV and peak head roll velocity, based on which gains of 0.28±0.09 in HU and 0.23±0.10 in the HD were found. No statistically significant differences emerged between HU and HD (p>0.125 for both). Individual eye movements data of the five participants whose head velocities were measured are presented in supplementary table 4.

### Re-test features

A subgroup of 4 participants (28.6%, 1 male, mean age 28.2±2.3), repeated the experiment with the difference of performing a longer duration head circumduction paradigm (59.9s versus 26.6s, as in the main paradigm). In the stopping response for these participants, the average duration of torsional nystagmus in the HU was 10.8±4.1s, with a TC of 3.5±1.5s (first test: 12.3±1.0s and 4.9±1.7s, respectively). In the HD average duration of response was 15.4±3.5s, with a TC of 5.7±0.2s (first test: 17.0±4.2s and 8.5±2.3s, respectively). The peak SPV in the HU post-rotational conditions was 32.9±10.5°/s, while in HD, the mean peak SPV was 20.8±7.9°/s (first test: 39.6±13.9°/s and 27.9±12.0°/s, respectively).

No statistically significant differences emerged when comparing the first test and re-test torsional nystagmus features in these participants (p>0.125 for all comparisons).

## Discussion

### Key findings

In this study, we aimed to validate the head circumduction maneuver as a paradigm to stimulate the torsional VOR, which is less-explored as compared to the extensively studied horizontal VOR. This is due largely to the need for specialized roll rotational equipment. Some current gaps in knowledge regarding torsional VOR include the influence of head reorientation on torsional nystagmus decay and the potential velocity storage mechanism role in the torsional plane. By developing a simpler method that does not require complex instrumentation, we sought to provide a new tool for investigating torsional VOR and exploratively address these issues. In the supplementary materials we provided a table summarising relevant previous torsional VOR paradigms and their main results (Supplementary table 5).

The main findings of our study may be summarized as follows:

1) This head circumduction paradigm, a simple and well tolerated manoeuvre, is successful in inducing a robust post-rotational torsional nystagmus. To our knowledge, this is the first potentially clinically applicable paradigm that induces such a consistent torsional VOR stopping response.
2) We assessed the role of otolith-vertical SCCs interaction on the time decay of the torsional nystagmus, which revealed that the forward head tilting prolonged the duration of response. This implies the presence of otolith dumping, and hence a velocity storage mechanism, in the torsional plane.
3) Most participants also showed a bilateral vertical nystagmus, which was either down-beating and disconjugate or skewed.
4) Horizontal nystagmus was minor and inconsistent across participants, strengthening the fact that this paradigm is optimal for stimulating the vertical SCCs, but not the horizontal canals.

The novelty of our paradigm lies with the fact that no sophisticated equipment is needed. Despite this, our data are generally and reassuringly consistent with previous research. Indeed, previous rotational paradigms for roll stimulation found torsional TCs averaging from 4.5 to 7.6 seconds (5, 9), compared to values from 4.1 to 7.2 seconds in the present study. Torsional gain was also reported to be low in other studies, ranging from 0.21 to 0.6 in the dark (0.26 to 0.28 in our case) across different paradigms (3, 5, 9, 15). This contrasts with gains ranging from 0.64 to 0.74 for the horizontal and vertical angular VOR (3). Moreover, previous studies found vertical eye disconjugacy induced by vertical SCCs stimulation, both during actual roll rotational tasks (14, 15) and on stopping responses (9), against the established belief that skew deviation of the eyes implies an otolith driven phenomenon (26–28).

There are several key aspects about our findings. First, the torsional VOR stimulation induced by the paradigm is robust. Indeed, the torsional nystagmus was not only present in all participants, but was also much stronger than vertical and horizontal components. The consistency of response to our simple paradigm means that this head movement could be used not only in research but also as a clinical test of torsional VOR function. The consistency of response duration and time constant even with a prolonged rotational paradigm (59.9s), which we observed in the subset of participants who repeated the task, confirmed that the 26.6s second circumduction task is enough to generate a stable response.

### Torsional otolith dumping

**Figure 7:**
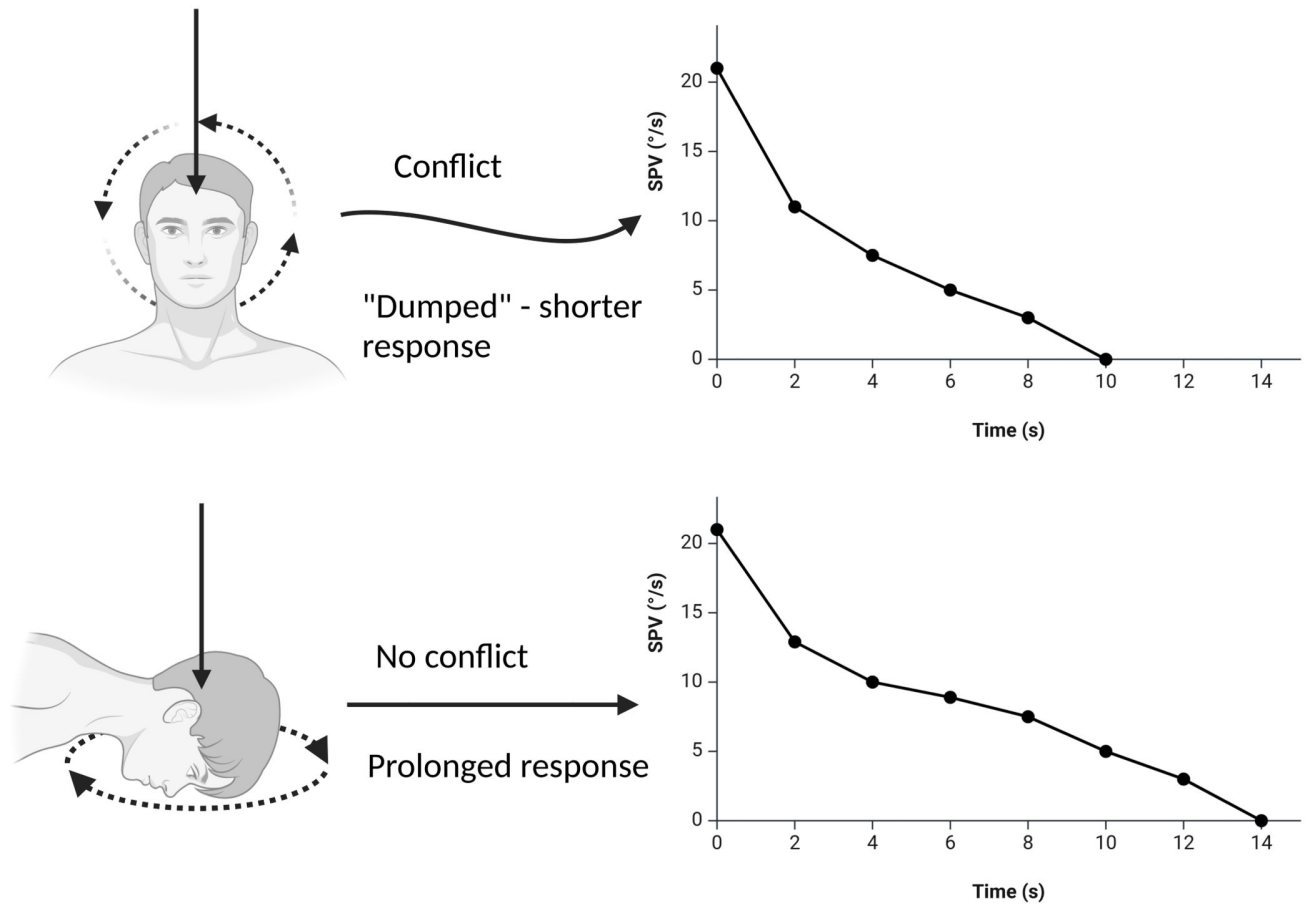
Schematic illustration of otolith-canal conflict in the torsional plane. Stopping response after stimulation of the vertical canals. The vertical canals activity is interpreted as rolling towards one side. Top graph: when the head is in an upright, neutral position, dynamic vertical canals activity should be in conflict with the static grativo-inertial signal generated by the otoliths, which may lead to the “otolith dumping”, i.e. shortening of nystagmus response. Bottom graph: if the head is forward tilted, rolling is not in conflict with the otoliths’ driven grativo-inertial signal, thus limiting the conflict between otoliths and semicircular canals activity. Legend: SPV = slow-phase velocity; °/s = degrees/second.

Our findings demonstrated, for the first time in humans, evidence of otolith dumping in the torsional VOR. Notably, forward head tilting during the stopping response prolonged nystagmus duration by on average 46.7%. This finding may represent an indirect measure of vertical semicircular canal velocity storage mechanism, which was thought to be of limited/no role in humans (5, 9). Interestingly, a previous study performed on monkeys (7) similarly found that in the stopping response the torsional TC of the animals was shorter with the head upright (i.e., in a neutral position) compared to when forward or backward tilted in the pitch plane. The shortening of torsional response with head up neutral indicates that this position elicits a conflict between the otolith input (signaling no rotation) and vertical SCCs activity (signaling rotation), which is resolved with a suppression of the post-rotational response through the velocity storage mechanism (8). This phenomenon has been called “tilt suppression” (11) because, being studied in the yaw plane-elicited horizontal nystagmus, the post-rotational forward head tilt shortens the response, in contrast to what we see here in the torsional plane (Figure 7). Indeed, for the horizontal SCCs, tilting the head in pitch creates a conflict between the input from the otoliths and the canals. In contrast, for the vertical (torsional) SCCs, this conflict occurs when the head is in a neutral, upright position (8, 10). An intriguing clinical implication of this paradigm rests in its possible role in assessing the velocity storage mechanism in patients with cerebellar nodulus/uvula lesions, who have shown an impaired tilt suppression of the horizontal nystagmus in previous research (11). Finally, head tilt also modulated the torsional peak SPV, a finding lacking a definitive explanation, although previous research noticed a similar trend for horizontal and vertical eye movements (29).

### Vertical canals induced eye disconjugacy

The present work confirms that vertical SCCs stimulation can induce eye disconjugacy in the vertical plane (9, 14, 15). Since earlier reports, the combination of skew deviation and ocular tilt was thought to be a clinical sign generated by otolith imbalance (26, 27). Indeed, this vertical misalignment can be typically elicited by static head tilts in the roll plane, suggesting a contribution of the otolithic tone (28, 30). However, static roll tilts with respect to gravity were not induced in our study participants, as well as in the previous reports of vertical disconjugacy elicited by low frequency rotational stimuli (14, 15). These data suggest a significant vertical SCCs contribution in inducing skew deviation. Interestingly, the change in otolithic input between the two post-rotational head conditions did not influence the degree of vertical disconjugacy, both in our study and in previous research (14). Why we observed inter-subject variability in the vertical nystagmic response is not clear, but possibilities include anatomical differences in the orientation of the vertical canals, minor subclinical differences in vestibular function amongst the subjects and variability in the motion stimuli as these were self-paced. However, it must be stressed that previous research conducted with passive stimuli in subjects strapped to precision rotatory mechanical devices produced identical vertical disconjugate and skewed eye movement pattern (9, 14, 15).

### Head trajectory and vestibular stimulation

**Figure 8:**
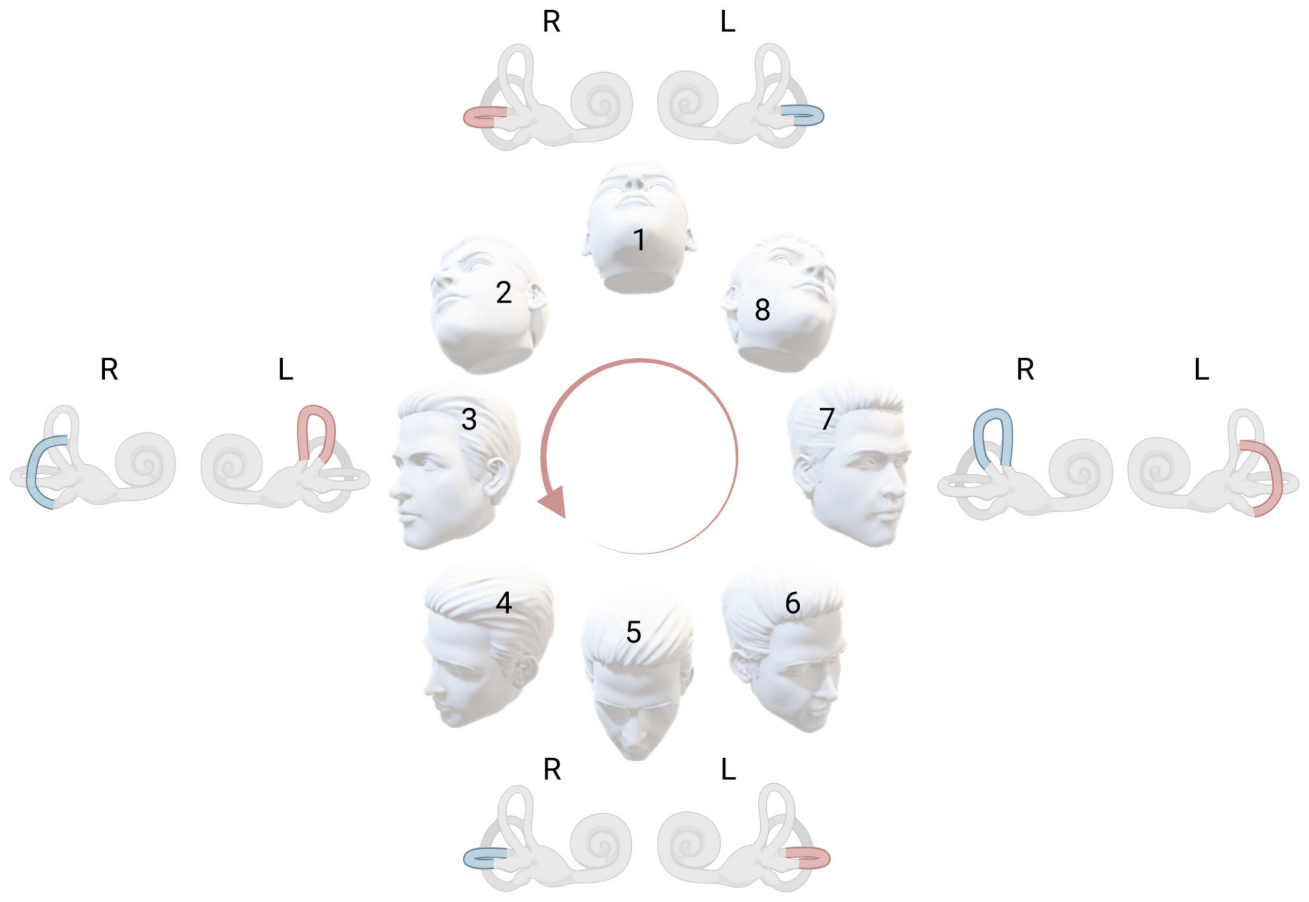
Schematic representation of the hypothesized stimulated/inhibited semicircular canals during a right circumduction. The stimulated left (L) and right (R) semicircular canals (SCC) during each manoeuvre’s steps are highlighted in red, and the inhibited in blue. Starting from the top (1) the first head movement is towards the top-right (2), which would mainly stimulate the right horizontal SCC. The downward movement from 2 to 4 is a left anterior SCC stimulating manoeuvre. From bottom-right (4) to bottom-left (6) is mainly a left horizontal SCC stimulating movement, and the upward movement from bottom-left to top-left (8) is a left posterior SCC stimulating movement, which terminates one cycle. Note that every cycle of right circumduction would cause a net stimulation of the left vertical canals. The opposite would happen for left circumduction, justifying the post-rotational nystagmic responses observed.

The complex nature of the circumduction head motion makes challenging to clearly deconstruct how the post-rotational response is generated. However, we can discuss some likely involved mechanisms. First, the presence of a right beating torsional nystagmus after a right circumduction task is apparently counterintuitive, since one could expect an opposite response. However, decomposing the head motion, right circumduction would mainly stimulate the left vertical SCCs. Indeed, when going from top to bottom with the head turned right, the main stimulated SCC is the left anterior, and the bottom-up movement while the head is turned to the left is a left posterior SCC stimulating maneuver, according to the left-anterior/right-posterior paradigm (31, 32). We show a schematic example of circumduction and activated semicircular canals for each step in figure 8. Additionally, our inertial sensor data showed that during a right circumduction there is a continuous, frequency modulated, roll velocity activity toward the left (Figure 6), while the opposite happens during left circumduction. These considerations suggest that what we call right circumduction is functionally like rolling toward the left for the participants, and the opposite for the leftward circumduction, justifying our eye movement results.

### Limitations

Our study has some limitations, mainly related to the self-paced nature of this circumduction motion. Indeed, it is not possible to fully control its velocity and trajectory. We accounted for this limitation by minimizing variability with several strategies: we carefully instructed and provided feedback to the study subjects, we used a metronome to control subjects’ pace and continuously monitored via infrared camera their execution throughout the study. Also, every task was performed in a randomized order, to minimize a possible additional learning effect. The symmetry of our findings and the consistency of the data in the subjects who performed a re-test several weeks later strengthen the impression that we reasonably controlled this variability. Additionally, we should acknowledge that comparison with other torsional VOR studies could only be made indirectly, for the intrinsic variability of the self-paced movement, the stimulation of other structures such as neck proprioceptive receptors and for being self-paced rather than passive. Indeed, active rotational strategies modulate both VOR gain and the velocity storage dependent phenomena (33, 34), which needs to be acknowledged when comparing our findings with previous literature. This paradigm, although simple, may not be performed by every subject, either for the coexistence of cervical disease or for the inability to understand the motion, which happened to one subject in our screening phase. Finally, although we performed a comprehensive clinical oculo-vestibular assessment, the lack of thorough instrumental vestibular screening might have allowed us to include participants with minor, subclinical vestibular dysfunction.

## Conclusion

We validated a novel stimulation paradigm for the torsional VOR, which does not require complex rotational equipment. This paradigm can be thought as an experimental model for inducing transient unilateral vertical SCCs asymmetry. Prospectively, this paradigm could be used to further explore peripheral and central processing of the vertical semicircular canals, both in healthy people and people affected by neurological and/or vestibular disorders.

## Supplementary Material

Supplementary tables and figures

## Figures and Tables

**Figure 9:**
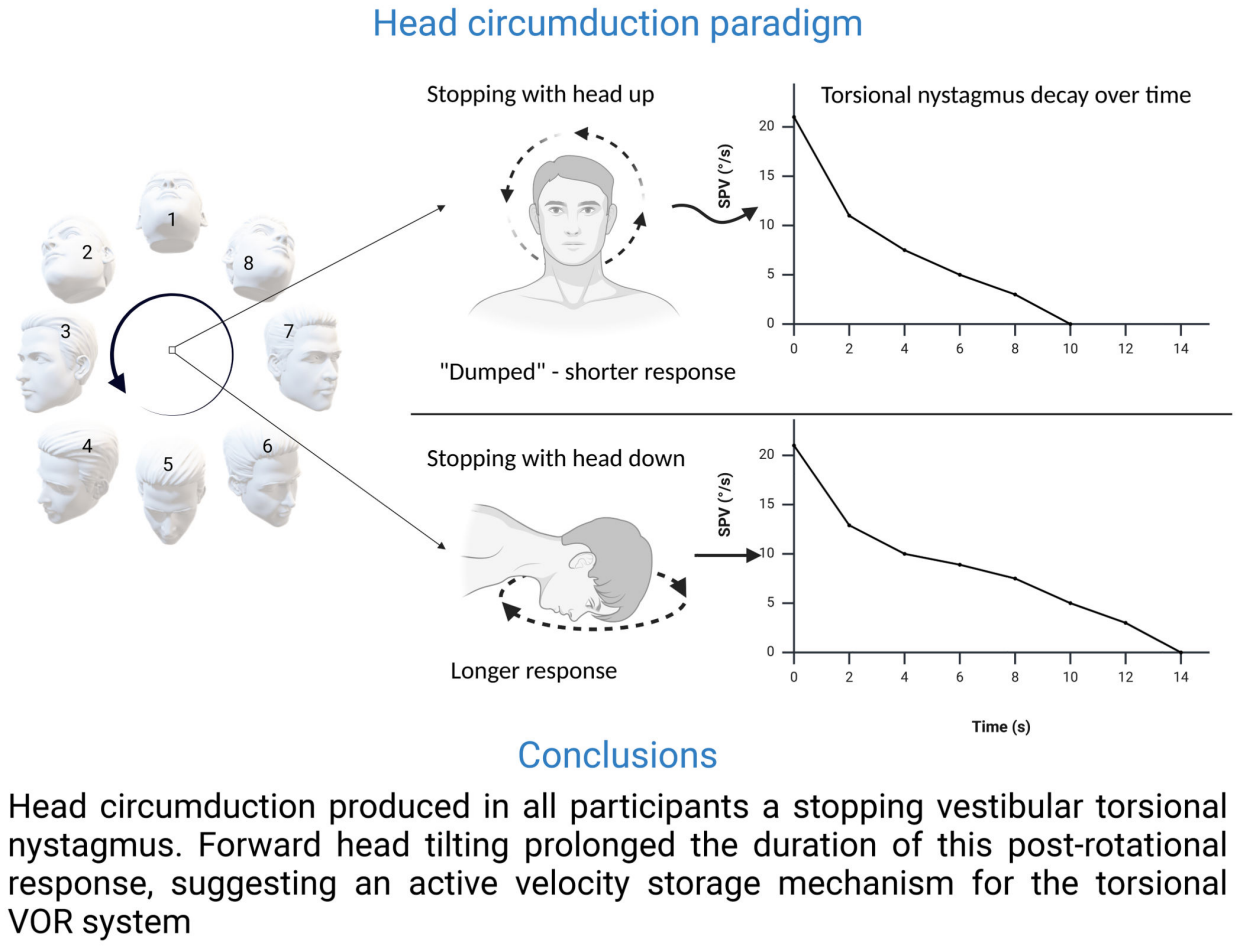


**Table 1:** Demographic features of the study population

	Male	Female	Total
Participants, n (%)	4 (28.6%)	10 (71.4%)	14
Mean age, years	28.5±3.4	26.8±6.0	27.3±5.3
Symptoms on stopping, n (%):			
- Non-spinning vertigo - Spinning vertigo - Nausea	- 4 (100.0%) - 0 (0.0%) - 1 (25.0%)	- 7 (70.0%) - 3 (30.0%) - 3 (30.0%)	- 11 (78.6%) - 3 (21.4%) - 4 (28.6%)
Change in HD, n (%):			
- Improvement - Worsening - No change	- 1 (25.0%) - 1 (25.0%) - 2 (50.0%)	- 8 (80.0%) - 1 (10.0%) - 1 (10.0%)	- 9 (64.3%) - 2 (14.3%) - 3 (21.4%)

Values are numbers ± standard deviation and % of total. Legend: HD = head down condition.

**Table 2:** Post-rotational vertical nystagmus features

	Participants without skew response (n=10)		*P* Value	Participants with skew response (n=3)		*P* Value
	Head up	Head down		Head up	Head down	
EE peak SPV (°/s)	8.9±4.7	9.1±4.3	0.820	5.2±6.1	5.4±4.5	0.750
IE peak SPV (°/s)	3.6±3.5	4.7±3.0	0.363	-7.3±5.5	-5.2±1.8	0.750
Cumulative SPD (°)	14.4±7.5	16.8±9.7	0.301	35.7±39.8	45.5±34.6	0.750

Values are means ± SD. Paired comparisons made between the two conditions by Wilcoxon signed-rank test. Legend: EE = extorting eye (upper pole going toward the temporal side during the fast phase), IE = intorting eye (upper pole going toward the nasal side during the fast phase), SPV = slow-phase velocity, SPD = slow-phase divergence, °/s = degrees/seconds, ° = degrees.

## Data Availability

Study data are available by contacting the corresponding author upon request.

## New & Noteworthy

Of all the tri-axial vestibulo-ocular reflexes, torsional is the least studied for the need of heavy-duty rotational equipment. We introduce a simple self-paced paradigm to study the torsional vestibulo-ocular reflex – head circumduction, which produced in all participants stopping vestibular torsional nystagmus when circumduction was stopped. Forward head tilting significantly prolonged the post-rotational torsional nystagmus duration – an ‘otolith dumping effect’ that suggests, for the first time in humans, a role of the velocity storage mechanism in the torsional vestibulo-ocular reflex.
