# Supplementary material for: Torsional nystagmus and otolith dumping effects investigated by head circumduction
Source: J Neurophysiol. Author manuscript; Available in PMC 2025 Jul 28. (PMC7617948; doi:10.1152/jn.00570.2024)
Supplement: Supplementary tables and figures [file EMS206323-supplement-Supplementary_tables_and_figures.docx]

**Supplementary table 1.** Post-rotational torsional nystagmus features by side of circumduction

|  | **Right circumduction** | |  | **Left circumduction** | |  |
| --- | --- | --- | --- | --- | --- | --- |
|  | **Head up** | **Head down** | ***P* Value** | **Head up** | **Head down** | ***P* Value** |
| Duration (s) | 9.8±3.1 | 14.5±4.2 | <0.0005 | 11.6±3.0 | 16.9±5.6 | <0.0005 |
| Time constant (s) | 3.5±1.3 | 6.8±2.8 | <0.0005 | 4.6±1.2 | 7.5±2.7 | <0.0005 |
| Peak SPV (°/s) | -31.0±15.0 | -22.8±13.9 | 0.0420 | 27.8±14.1 | 19.3±7.4 | 0.0070 |

**Supplementary table 1.** Values are means ± SD. Paired comparisons made between the two conditions by Wilcoxon signed-rank test. Legend: s = seconds, °/s = degrees/seconds.

**Supplementary table 2.** Post-rotational vertical nystagmus features by side of circumduction

|  | **Subjects without skew response** | | | | |  | **Subjects with skew response** | | | | |  |
| --- | --- | --- | --- | --- | --- | --- | --- | --- | --- | --- | --- | --- |
|  | **Right circumduction** | |  | **Left circumduction** | |  | **Right Circumduction** | |  | **Left circumduction** | |  |
|  | **Head up** | **Head down** | ***P* Value** | **Head up** | **Head down** | ***P* Value** | **Head up** | **Head down** | ***P* Value** | **Head up** | **Head down** | ***P* Value** |
| RE peak SPV (°/s) | 10.3±7.4 | 8.5±5.2 | 0.947 | 3.0±1.7 | 5.4±4.9 | 0.195 | 3.1±2.8 | 5.0±4.7 | 0.500 | -7.7±7.8 | -4.2±2.8 | 1.000 |
| LE peak SPV (°/s) | 4.2±5.6 | 4.9±3.1 | 0.673 | 7.5±4.2 | 9.9±5.7 | 0.203 | -7.0±3.2 | -6.0±1.7 | 0.500 | 7.3±9.5 | 6.6±5.9 | 1.000 |
| Cumulative SPD (°) | 15.1±6.8 | 13.7±3.5 | 0.578 | 13.6±9.0 | 21.7±15.3 | 0.074 | 34.4±34.0 | 43.3±31.3 | 1.000 | 36.9±45.7 | 48.8±53.5 | 1.000 |

**Supplementary table 2.** Values are means ± SD. Paired comparisons made between the two conditions by Wilcoxon signed-rank test. Legend: RE = right eye, LE = left eye, SPV = slow-phase velocity, SPD = slow-phase divergence, °/s = degrees/seconds, ° = degrees.

**Supplementary table 3.** *Post-rotational horizontal nystagmus features by side of circumduction*

|  | **Subjects with horizontal nystagmus beating towards the same side of the torsional component (n=7)** | | | | | | **Subjects with horizontal nystagmus beating towards the opposite side of the torsional component (n=2)** | | | | | | **Subjects with post-rotational left beating nystagmus (n=3)** | | | | | | |
| --- | --- | --- | --- | --- | --- | --- | --- | --- | --- | --- | --- | --- | --- | --- | --- | --- | --- | --- | --- |
|  | **Right circumduction** | | | **Left circumduction** | | | **Right Circumduction** | | | **Left circumduction** | | | **Right Circumduction** | | | **Left circumduction** | | | |
|  | **Head up** | **Head down** | ***P* Value** | **Head up** | **Head down** | ***P* Value** | **Head up** | **Head down** | ***P* Value** | **Head up** | **Head down** | ***P* Value** | **Head up** | **Head down** | ***P* Value** | **Head up** | **Head down** | ***P* Value** |  |
| Peak SPV (°/s) | -7.5±4.5 | -4.4±2.1 | 0.500 | 9.4±5.6 | 5.1±2.5 | 0.500 | 6.7±5.3 | 4.3±3.0 | 0.500 | -4.7±4.0 | -7.3±4.0 | 1.000 | 2.6±0.7 | 4.7±1.2 | 0.500 | 7.3±3.0 | 6.4±4.1 | 0.500 |  |

**Supplementary table 3**. Values are means ± SD. Paired comparisons made between the two conditions by Wilcoxon signed-rank test. Legend: SPV = slow-phase velocity, °/s = degrees/seconds.

**Supplementary table 4.** Torsional individual eye movements data of the five participants whose head velocities were measured.

|  | **Head up** | | | | | **Head down** | | | | |
| --- | --- | --- | --- | --- | --- | --- | --- | --- | --- | --- |
| **Participant ID** | **Nystagmus duration (s)** | **Time constant (s)** | **Peak SPV (°/s)** | **Peak head roll velocity (°/s)** | **Gain** | **Nystagmus duration (s)** | **Time constant (s)** | **Peak SPV (°/s)** | **Peak head roll velocity (°/s)** | **Gain** |
| 1 | 11.1±1.6 | 3.1±0.8 | 46.5±25.0 | 216.6±20.6 | 0.21±0.13 | 11.4±1.9 | 5.7±1.6 | 31.0±13.9 | 222.8±53.0 | 0.14±0.10 |
| 2 | 12.0±3.8 | 6.7±1.1 | 19.0±9.0 | 98.8±4.7 | 0.20±0.10 | 16.4±6.0 | 10.8±2.3 | 16.2±7.1 | 128.1±52.9 | 0.13±0.12 |
| 3 | 12.8±3.7 | 3.1±0.7 | 24.3±2.3 | 57.7±28.2 | 0.42±0.37 | 18.9±5.8 | 5.8±1.9 | 23.0±7.3 | 61.9±28.8 | 0.37±0.23 |
| 5 | 13.4±4.8 | 6.1±0.5 | 49.4±4.1 | 176.8±49.4 | 0.28±0.10 | 21.1±0.9 | 7.6±1.3 | 43.4±9.7 | 154.6±41.2 | 0.28±0.02 |
| 9 | 10.7±0.7 | 3.7±0.5 | 17.7±10.2 | 59.8±10.5 | 0.29±0.01 | 13.0±2.8 | 5.3±0.4 | 14.6±2.8 | 55.3±7.5 | 0.26±0.12 |
| Total average | 12.0±1.1 | 4.6±1.7 | 31.4±15.3 | 121.9±71.6 | 0.28±0.09 | 16.2±4.0 | 7.0±2.3 | 25.6±11.8 | 124.5±69.4 | 0.23±0.10 |

**Supplementary table 4.** Values are means ± SD. Gain has been calculated as the ratio between peak SPV velocity and peak head roll velocity. Legend: s = seconds, °/s = degrees/seconds.

**Supplementary table 5.** Previous torsional vestibulo-ocular reflex stimulation paradigms

| **Study** | **Population** | **Stimulation** | **Eye evaluation frame** | **Torsional TC** | **Torsional gain** | **Comment** |
| --- | --- | --- | --- | --- | --- | --- |
| (Tweed *et al.*, 1994b) | One female and five male participants | Rotator-driven roll sinusoidal rotations at 0,3 Hz (37.5°/s) along earth-vertical and -horizontal axes in the dark | During the stimulus | N.A. | 0.37±0.09 (earth-vertical roll rotation) – 0.39±0.11 (earth horizontal roll rotation) | This is a seminal paper which assessed VOR gain across the horizontal, vertical and torsional planes during different axes of sinusoidal stimulations. Torsional gain was not significantly influenced by otolith-canal interaction (no difference between earth-vertical and horizontal roll stimulations) |
| (Tweed *et al.*, 1994a) | One female and four male participants | Rotator-driven roll constant velocity stimulation at 150°/s for 29 s along earth-vertical and -horizontal axes in the dark | During the stimulus | 7.3 s (earth vertical roll rotation) – 7.6 s (earth horizontal roll rotation) | 0.21±0.08 (earth-vertical roll rotation) – 0.23±0.07 (earth horizontal roll rotation) | The findings of this paper suggested the hypothesis that there is little to no velocity storage in the torsional/vertical plane, since TCs were similar to the vestibular nerve’s estimated TC |
| (Jauregui-Renaud *et al.*, 1998) | Two female and one male participants | Manual driven roll quasi-sinusoidal oscillation at 0.1 - 0.4 Hz (25.2±3.1°/s -72.3±4.7°/s) along earth-vertical or horizontal axes), in the dark or light | During the stimulus | N.A. | Between 0.3 and 0.6 in the dark, both along earth-vertical horizontal axes  Between 0.5 and 0.8 in the light | This paper confirmed, with a different stimulation paradigm, the findings regarding low gain in the torsional plane. Additionally, vertical disconjugate eye movement induced by roll stimuli were described. Torsional gain was not significantly influenced by otolith-canal interaction (no difference between earth-vertical and horizontal roll stimulations) |
| (Jauregui-Renaud *et al.*, 2001) | Three female and three male participants | Manually driven couch roll constant velocity stimulation at 56.5±3.1°/s for 40 s along earth-vertical axis in the dark | Stopping response without head reorientation | 4.5±1.5 s | 0.39±0.06 | This experiment confirmed the findings regarding short TC and low gain in the torsional plane evaluated during a stopping response. Disconjugate vertical eye movement were also noted |
| (Angelaki & Hess, 1994) | Six rhesus monkeys | Rotator-driven supine roll oscillation at 90°/s until exhaustion of per-rotatory response | Stopping response with and without head reorientation | 2.7±0.7 s (head upright neutral) - >15 s (head forward or backward tilted in pitch) | N.A. | This study performed on monkeys found a significant interaction between head reorientation in pitch plane, and change of post-rotational TC of torsional nystagmus, with the shortest being with the head upright |

**Supplementary table 5.** Summary of relevant previous studies assessing torsional VOR. Legend: TC = time constant, Hz = Hertz, ° = degrees, s = seconds, N.A. = not assessed, VOR = vestibulo-ocular reflex.

**Supplementary figure 1.** Representative subject’s pitch plane angular velocity during a right circumduction.


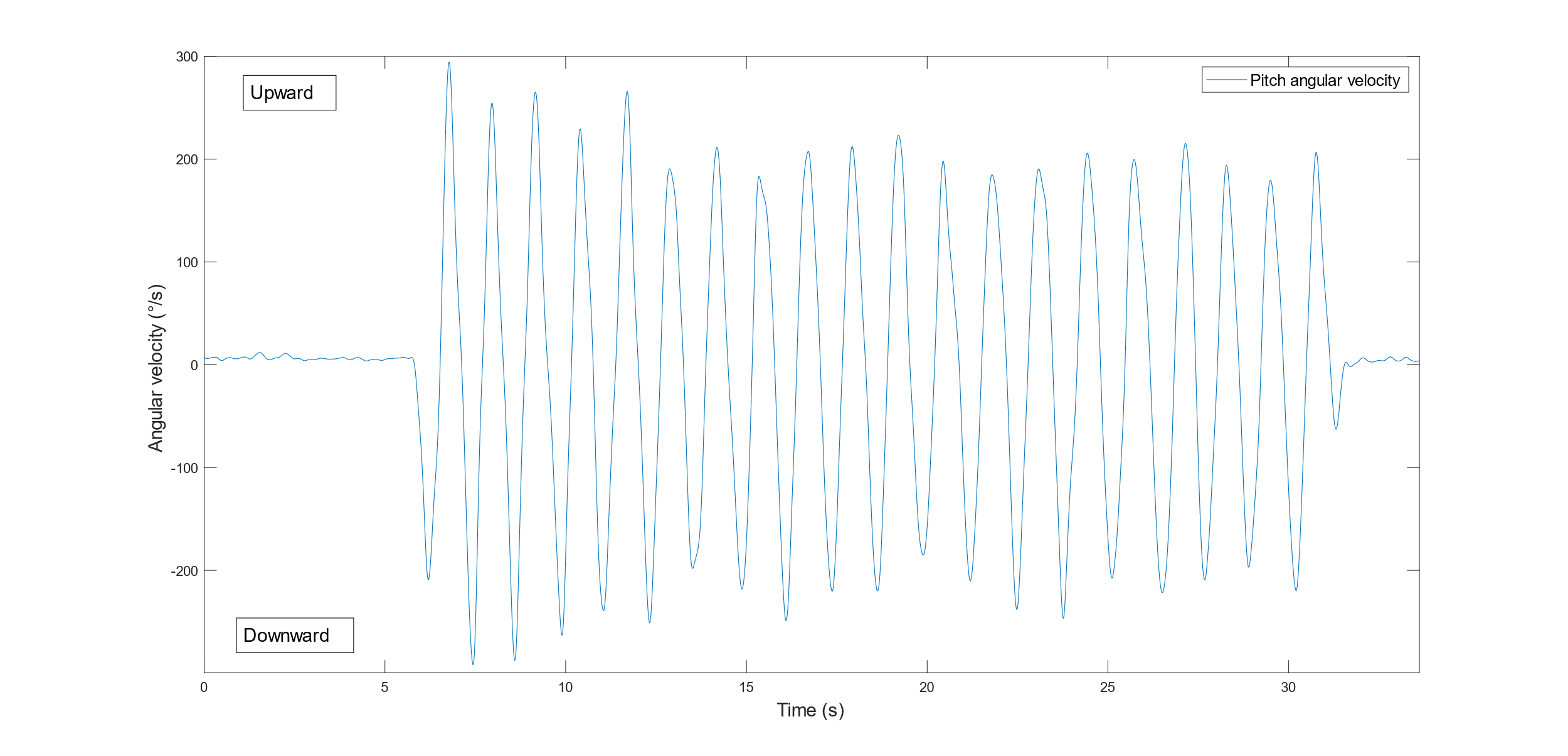


**Supplementary figure 1.** Representative subject's pitch plane angular velocity during a right circumduction. For the whole population, the mean peak upward pitch plane angular velocity during circumduction followed by HU condition was 183.1±34.9°/s, while peak downward velocity was -205.1±22.5°/s. The overall average pitch velocity (sampling all the velocities) was 0.8±5.0°/s. In trials followed by a HD stopping response, the peak upward velocity was 195.1±28.1°/s, while the peak downward velocity was -208.8±13.8°/s, the overall average velocity was -1.6±4.3°/s. Legend: HU = head up, HD = head down, °/s = degrees/seconds, s = seconds.

**Supplementary figure 2.** Representative subject’s yaw plane angular velocity during a right circumduction.
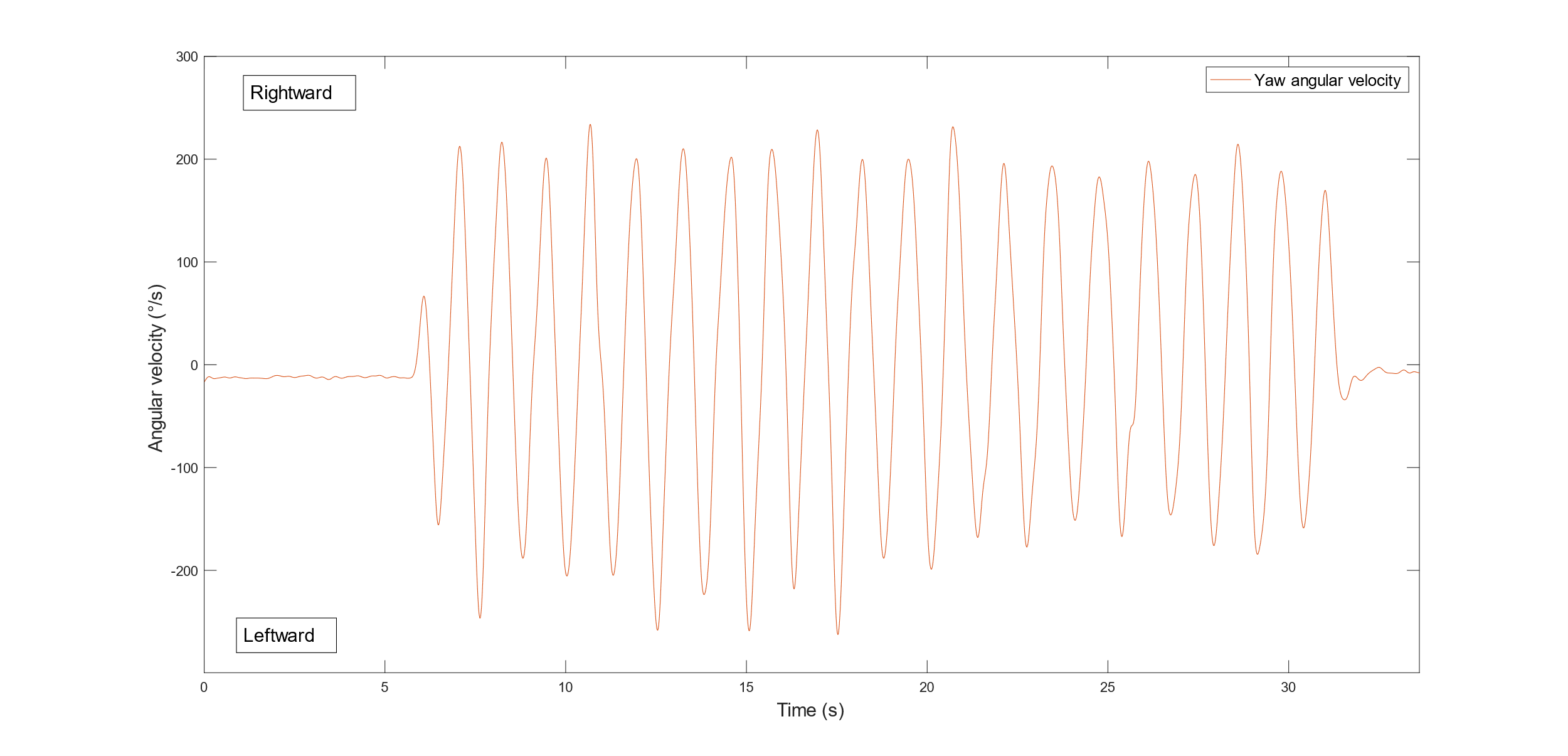


**Supplementary figure 2.** Representative subject's yaw plane angular velocity during a right circumduction. For the whole population, in trials followed by HU, the mean peak rightward velocity was 197.2±13.8°/s, while the peak leftward velocity was -222.8±31.1°/s, the overall average velocity was -1.7±5.9°/s. In trials followed by HD, the peak rightward velocity was 212.2±46.1°/s, while the peak leftward velocity was -237.4±55.1°/s, the overall average velocity was -1.8±4.4°/s. Legend: HU = head up, HD = head down, °/s = degrees/seconds, s = seconds.

**References**

Angelaki DE & Hess BJ. (1994). Inertial representation of angular motion in the vestibular system of rhesus monkeys. I. Vestibuloocular reflex. *J Neurophysiol* **71,** 1222-1249.

Jauregui-Renaud K, Faldon M, Clarke AH, Bronstein AM & Gresty MA. (1998). Otolith and semicircular canal contributions to the human binocular response to roll oscillation. *Acta Otolaryngol* **118,** 170-176.

Jauregui-Renaud K, Faldon ME, Gresty MA & Bronstein AM. (2001). Horizontal ocular vergence and the three-dimensional response to whole-body roll motion. *Exp Brain Res* **136,** 79-92.

Tweed D, Fetter M, Sievering D, Misslisch H & Koenig E. (1994a). Rotational kinematics of the human vestibuloocular reflex. II. Velocity steps. *J Neurophysiol* **72,** 2480-2489.

Tweed D, Sievering D, Misslisch H, Fetter M, Zee D & Koenig E. (1994b). Rotational kinematics of the human vestibuloocular reflex. I. Gain matrices. *J Neurophysiol* **72,** 2467-2479.
